# Editorial: Symmetry as a guiding principle in artificial and brain neural networks

**DOI:** 10.3389/fncom.2022.1039572

**Published:** 2022-10-24

**Authors:** Fabio Anselmi, Ankit B. Patel

**Affiliations:** ^1^Department of Neuroscience, Baylor College of Medicine, Houston, TX, United States; ^2^Center for Neuroscience and Artificial Intelligence, Baylor College of Medicine, Houston, TX, United States; ^3^Department of Electrical and Computer Engineering, Rice University, Houston, TX, United States

**Keywords:** neural networks, visual cortex, symmetry, perceptual constancy, robustness, smell, kernels, implicit regularization

Recent advances in neuroscience have brought unprecedented growth in the scale and complexity of data to be processed. In this scenario, it is crucial to understand the principles, constraints, and goals of neural computation to extract meaningful information. One such principle is symmetry. For example, in the vision domain, this corresponds to the ability to grasp the essential features of an object even if presented in a great variety of different aspects and viewing conditions (thought of as symmetries).

This Research Topic collects novel types of analysis and methods that use the principle of symmetry to shed light on: (1) how the brain may use symmetries to process and organize sensory information; (2) how modern artificial neural networks can implement and take advantage of such symmetries.

In the first article of the Topic, Pizlo and de Barros analyze **perceptual constancy** (i.e., the fact that the perceived characteristics of objects remain constant despite their transformations) and how it relates with symmetry. The authors argue that at the core of perceptual constancy lies the permanence of objects' **invariant features** through time (shape, size, and weight). Mental representations of sensory signals that rely on such invariants are considered “good” representations. In particular, using established mathematical results (Noether's theorems), they analyze the case of mirror-symmetrical objects deriving an invariant representation through the application of a simplicity (least-action) principle.

The idea that **“good” representations** could underlie the ability of biological intelligence to produce complex behavior through data efficient, generalizable, and transferable skill acquisition is also the main point of the review by Higgins et al. Evidence for such good representations in the cortex is provided and analyzed through the mathematical lens of **slow features learning** by the work of Rolls. He presents neurophysiological evidence for the learning of invariant representations in the inferior temporal visual cortex (object and face representations invariant to position, size, lighting, view, and morphological transforms), in the superior temporal sulcus (invariance to global object motion) and hippocampus (invariance to eye position, head direction, and place). He proposes a computational mechanism for the brain to learn these invariant representations based on slow features and unsupervised learning and implement it in a hierarchical artificial feed-forward architecture (VisNet).

Focusing on the concept of time, Betti et al., further extend the theory of good representation based on sensory input symmetries incorporating the dynamical constraints of motion coherence. They formulate a theory of the development of visual features based on the objects **optical flows** arguing that this new type of learning is key for low-sample complexity training from data video streams.

Finally, in the contribution of Krishnamurthy et al., the authors go beyond vision considering a model of the **smell** signal in the brain. They show how the neural processing of sparse but high-dimensional representation of the olfactory information differs from the other senses in its fundamental use of disorder.

The second group of contributions to the Topic focus on how artificial neural networks represent symmetries and how this is related to good network properties such as generalization and robustness.

Bertoni et al. propose a convolutional neural network architecture with biological constraints and show the emergence of **spontaneous symmetries**, analogously to those in the visual system, in the early layers during learning on natural images. Their architecture is composed of a pre-filtering step (in analogy with the Lateral Geniculate Nucleus) and a convolutional layer with lateral connections (in analogy with the horizontal connectivity of the primary visual cortex, V1). They provide evidence that the pre-filter evolves, during training, to reach a radially symmetric pattern well approximated by a Laplacian of Gaussian (LoG), a well-known model of the receptive profiles of LGN cells. Further, they show that the learned convolutional filters in the first layer can be approximated by Gabor functions together with orientation selectivity and horizontal connections in agreement with well-established models for the receptive profiles of V1 simple cells.

The contributions described so far mainly focus on the invariant properties of the sensory data representation. However, besides invariance, **equivariance** (i.e., how the representation changes upon transformations of the network's input) is also a key property of modern artificial neural networks. This property and its extensions are studied in the topic contribution by Conti et al. where the authors propose Group Equivariant Operators as fundamental objects to built equivariant networks with low sample complexity.

The work of Sahs et al. takes another point of view and analyzes the interplay between the network weights symmetries and its **implicit regularization** i.e., the bias of the network's input representation due to its architecture, weights, initialization and optimization algorithm. They consider two types of symmetry transformations: permutation of the neurons and rescaling of weight and bias parameters. Using the theory of splines they develop a transparent view of the structure of the loss surface associated with the task, including its critical and fixed points, Hessian, and Hessian spectrum regularization.

A more explicit analysis of the implicit regularization in terms of the **essential frequencies** of an image needed by a network to achieve a good classification performance is provided in Karantzas et al. The authors show how, surprisingly, very few image frequencies are needed to achieve generalization and robustness of the network and how this constitutes a fingerprint of the implicit bias of the network.

Finally, the work of Barbieri takes a more classical signal processing point of view and considers the problem of reconstructing an image that is downsampled in the space of a wavelet transform based on the *SE*(2) group. Using such computational framework, which is motivated by classical models of simple cell receptive fields in the primary visual cortex, he proves that the solution relies on the **reproducing kernel** arising from the *SU*(2)-symmetry group structure.

We hope that the reader will find this Research Topic useful in understanding how symmetry plays a fundamental role in shaping the way a biological and artificial neural network processes information. In particular how a neural network can use symmetry to achieve a “good” representation i.e., with good generalization, robustness, and low sample complexity properties.

## Author contributions

FA and AP wrote the editorial. Both authors contributed to the article and approved the submitted version.

## Conflict of interest

The authors declare that the research was conducted in the absence of any commercial or financial relationships that could be construed as a potential conflict of interest.

## Publisher's note

All claims expressed in this article are solely those of the authors and do not necessarily represent those of their affiliated organizations, or those of the publisher, the editors and the reviewers. Any product that may be evaluated in this article, or claim that may be made by its manufacturer, is not guaranteed or endorsed by the publisher.

